# Engineering CD5-targeting CAR-NK cells from peripheral blood for the treatment of CD5-positive hematological malignancies

**DOI:** 10.1186/s12967-025-06432-3

**Published:** 2025-04-08

**Authors:** Haolong Lin, Lingfeng Zhang, Tong Ge, Ning An, Yongkun Yang, Yicheng Zhang, Wei Mu

**Affiliations:** 1 Department of Hematology, Tongji Hospital, Tongji Medical College, Huazhong University of Science and Technology, 1095 Jiefang Avenue, Wuhan, 430030 Hubei P. R. China; 2 Immunotherapy Research Center for Hematologic Diseases of Hubei Province, Wuhan, 430030 Hubei China; 3 Nanjing IASO Biotherapeutics Ltd, Nanjing, China

**Keywords:** Cellular immunotherapy, CAR-NK, CD5, Hematologic malignancies

## Abstract

**Background:**

The therapeutic application of chimeric antigen receptor (CAR) T cells in T-cell malignancies faces substantial limitations owing to fratricide and potential T cell aplasia, primarily attributed to the shared expression of target antigens, such as CD5, between normal and malignant T cells. Although natural killer (NK) cell-based immunotherapy is a promising alternative approach, its efficacy in treating hematologic malignancies remains to be fully elucidated.

**Methods:**

CD5-targeted CAR-modified primary NK cells, T cells and NK92 cell lines were generated and comprehensively evaluated for their anti-tumor efficacy through in vitro cytotoxicity assays and xenograft mouse models. Furthermore, preliminary investigation of the herpes simplex virus-1 thymidine kinase (HSV-TK) suicide switch system in CAR-NK cells were conducted using ganciclovir (GCV) as the activating agent.

**Results:**

CAR-NK cells exhibited significantly increased cytotoxic activity against CD5-positive cell lines and primary tumor cells, compared to NK, CAR-NK92, and CAR-T cells. Moreover, CAR-NK cells effectively decreased the leukemic burden and extended survival in murine model. Additionally, an off-switch utilizing the HSV-TK switch system successfully eradicated CAR-NK cells for safety considerations.

**Conclusions:**

This study developed a controllable CD5 CAR-NK cells that exhibit high efficacy against T-cell malignancies, although further validation is necessary to assess their clinical potential.

**Supplementary Information:**

The online version contains supplementary material available at 10.1186/s12967-025-06432-3.

## Introduction

T cell malignancies are a group of hematological cancers with complex genetic heterogeneity [1, 2]. The overall prognosis of refractory or relapsed (r/r) T-cell malignancies is poorer than that of B-cell malignancies, with fewer curative options available [3]. Novel cellular immunotherapy strategies employing chimeric antigen receptor (CAR)-modified T cells have identified CD5 as a promising therapeutic target for T-cell malignancies [4, 5] and other CD5-expressing hematologic malignancies, such as diffuse large B-cell lymphoma (DLBCL) and chronic lymphocytic leukemia (CLL) [6, 7]. However, its clinical application is hindered by the following reasons: (1) potential contamination of patient T cells with tumor cells [8, 9], (2) T cell dysfunction/deficiency in patients, (3) Fratricide of CD5 targeting CAR-T cells during the manufacturing process [5, 10], (4) Potential immunodeficiency caused by prolonged T cell aplasia.

Natural killer (NK) cells, a crucial component of the innate immune system, inherently lack CD5 surface antigens [11]. This characteristic makes them ideal candidates for genetic modification of CAR-expressing NK cells, which can be utilized as targeted immunotherapy against CD5-positive hematological malignancies. The “off-the-shelf” NK cells can be derived from the peripheral blood (PB), cord blood (CB) and cell lines such as NK92. It is well established that CB-derived NK cells are immature and less cytotoxic than PB-derived NK cells [12]. Immortalized NK cell lines are typically irradiated before infusion, which limits their proliferation and cytotoxic capacity in vivo [13]. PB-NK cells obtained from healthy allogeneic donors have demonstrated significant therapeutic potential in the treatment of r/r acute myeloid leukemia (AML), high-risk myelodysplastic syndromes (MDS), and various other malignancies [14–16]. Moreover, CAR-NK cell therapy has exhibited considerable clinical efficacy and superior safety in clinical settings compared with conventional treatments [17, 18]. Recent clinical trials have substantiated both the therapeutic efficacy and enhanced safety of these cellular immunotherapy approaches. These encouraging results provide momentum for the development of PB-derived CD5 CAR-NK cells as a promising therapeutic option for CD5-positive hematological malignancies.

In this study, anti-CD5 heavy-chain variable (VH) domains incorporated into the CAR design were isolated from a fully human phage display library, which has demonstrated robust performance in previous studies [5, 19]. To address the safety concerns associated with CD5 CAR-T cell therapy, we incorporated a suicide gene system into CD5 CAR-NK cells. The herpes simplex virus thymidine kinase (HSV-TK)/ganciclovir (GCV) system [20], a well-established safety switch mechanism, was integrated into the CAR-NK cell design. This suicide gene strategy enables rapid elimination of engineered cells upon administration of GCV, providing a crucial safety measure to mitigate potential adverse events during treatment. A comprehensive comparison of the tumor-killing capacity was conducted among CD5-targeting CAR-engineered NK cells, T cells and NK92 cell lines. CD5 CAR-NK cells exhibited significantly higher cytotoxicity against malignant cell lines, primary leukemic cells, and mouse xenograft tumors. While on-target off-tumor cytolysis of normal T cells remains an inherent challenge, the administration of low-dose GCV effectively eliminates CAR-NK cells co-expressing the suicide gene switch. Our findings provide compelling proof-of-concept evidence supporting the therapeutic potential of CD5 CAR-NK cell therapy for targeting CD5-positive hematological malignancies. Furthermore, our comprehensive evaluation validated the therapeutic efficacy of this approach and established a robust foundation for future clinical investigations.

## Methods

### Cell culture

Jurkat, CCRF-CEM, Molt-4 and SUP-T1 cells are CD5-positive, CD5 knockout CCRF-CEM (CD5KO-CCRF) cell line was generated as previously described [5]. All of these are derived from acute T cell leukemia or T cell lymphoma. K562 (chronic myeloid leukemia cells) and Raji (Burkitt’s lymphoma) are CD5 negative cell line that was used as control. These cells were engineered to express firefly luciferase by transfection with lentiviral vectors and were cultured in RPMI-1640 medium (Thermo Fisher Scientific, Waltham, MA, USA) containing 10% fetal bovine serum (FBS) (Thermo Fisher Scientific, Waltham, MA, USA). HEK293 cells were cultured in DMEM (Thermo Fisher Scientific, Waltham, MA, USA) supplemented with 10% fetal bovine serum (FBS). NK92 cells were cultured in α-MEM (Thermo Fisher Scientific, Waltham, MA, USA) supplemented with 12.5% FBS, 12.5% horse serum (Thermo Fisher Scientific, Waltham, MA, USA), 200 IU/mL IL-2 (BeijingShuanglu Inc, Beijing, China) and 100 µM β-mercaptoethanol (Thermo Fisher Scientific, Waltham, MA, USA).

### Patient samples

Frozen primary leukemia cells were thawed and cultivated in RPMI-1640 medium containing 10% FBS and 200 IU/ mL IL-2 (detailed information for each patient is provided in Table S1). The use of primary patient material was approved by Ethic Commission of Tongji Hospital, Huazhong University of Science and Technology (Approval ID: TJ-IRB202406058). All the participants provided written informed consent in accordance with the Declaration of Helsinki.

### CAR construction and lentiviral production

Second-generation CAR structures incorporating two VH domains were constructed. Briefly, VH was combined in frame with the CD8 hinge, transmembrane domain, 4 − 1 BB costimulatory domain and CD3ζ activation domain. Secretory IL15 was included to facilitate CAR-NK cells proliferation. The HSV-TK gene, serving as a suicide gene safety switch, was co-constructed with the CAR sequence in the plasmid vector No. 2504. Third-generation baboon envelope-pseudotyped lentiviral vectors were produced by transfection into HEK293T cells using Lipo3000 (Thermo Fisher Scientific, Waltham, MA, USA) [21].

### Manufacturing of CD5 CAR-NK, CD5 CAR-NK 92 and CD5 CAR-T cells

Peripheral blood mononuclear cells (PBMCs) for CAR-NK and CAR-T cell generation were purchased from Milestone Biological Science&Technology Co., Ltd. (Shanghai, China). CD5 CAR-NK and CAR-T cells were manufactured as previously described [5, 22]. Briefly, NK cells were isolated according to the manufacturer’s instructions using an NK cell isolation kit (Miltenyi Biotec, Bergisch Gladbach, Germany) and cultured in KBM581 medium (Corning, New York, USA) supplemented with 10% FBS and 200 IU/ mL IL-2. Fresh NK cells were activated using m21-K562 feeder cells (Hangzhou Zhongying Biomedical Technologies, China) at a ratio of 1:2. Activated NK cells were transducted with lentivirus at a multiplicity of infection (MOI) of five on Day 4. T cells were isolated using CD3 microbeads (Miltenyi Biotec, Bergisch Gladbach, Germany) according to the manufacturer’s instructions and then cultured in X-VIVO 15 medium (Lonza, Basel, Switzerland) supplemented with 10% FBS and 200 U/mL IL-2. Fresh T cells were activated with Dynabeads™ Human T-Activator CD3/CD28 (Thermo Fisher Scientific, Waltham, MA, USA). CD5 gene disruption was performed 24 h after T cell activation, followed by lentiviral transduction which was completed over the subsequent 24-hour period.

### Flow cytometry analysis

CD5, HLA-I, HLA-II, MICA/MICB and ULBPs expression in tumor cell lines as well as primary tumor cells were analyzed by flow cytometry using PE-anti-CD5 (clone UCHT2, BioLegend), APC-anti-HLA-A/B/C (clone W6/32, BioLegend), APC-anti-HLA-DP/DQ/DR (clone Tü39, BioLegend), APC-anti-MICA/MICB (clone 6D4, BioLegend) and Alexa Fluor 488-anti-ULBPs (clone 165903, R&D) respectively. FITC-labeled Human CD5 protein (CD5-H52H2, Acro) was used to detect the CAR expression. The purity and subtype of NK cells were identified using percp-anti-CD45 (clone: HI30, BioLegend), APC-anti-CD56 (clone: HCD56, BioLegend), PE-anti-CD3(clone: HIT3a, BioLegend), and BV605-anti-CD16 (clone: 3G8, BioLegend). The following antibodies were used to analyzed the immunotype of CAR-NK cells: BV650-anti-CD69 (clone: FN50, BioLegend), PE-anti-CD96 (clone: NK92.39, BioLegend), PE-anti-NKp30 (clone: P30-15, BioLegend), Pacific Blue -anti-NKp46 (clone: 9E2, BioLegend), APC-anti-NKG2A (clone: S19004C, BioLegend), APC-Cy7-anti-NKG2D (clone: 1D11, BioLegend), BV421-anti-PD-1 (clone: EH12.2H7, BioLegend), and BV605-anti-TIGIT (clone: A15153G, BioLegend). Staining was performed according to the manufactures’ instructions.

### CD107a degranulation assay

CAR-NK, CAR-T, CAR-NK92, and NK cells were co-cultured with different cell lines or healthy donor (HD)-derived T cells at a 1:1 ratio. PE/Cyanine7-anti-CD107a antibody (clone: H4A3; Biolegend) and monensin solution (BD Biosciences, New Jersey, USA) were added to each well. The cells were then incubated at 37 °C with 5% CO2 for 4 h and analyzed using flow cytometry.

### In vitro cytotoxicity assays

CAR-NK, CAR-T, CAR-NK92, NK cells, and luciferase-expressing cell lines were seeded in triplicate at indicated ratios and incubated at 37 ℃ in 5% CO_2_ for 4 h. After incubation, steady-Glo luciferase substrate (Promega, Madison, WI, USA) was added and the plate was kept away from light, and the luminescence was measured using a BioTek synergy2 10 min later. The results are reported as percent killing based on the luciferase activity in wells with target cells but no effector cells (% killing = 100 – ((relative light unit (RLU) from wells plated with effector and target cells) / (RLU from wells plated with target cells only) ×100).

Cytolysis of primary tumor cells and HD T cells was determined by flow cytometry. Briefly, primary cells from different patients and healthy donors were labeled with Cell Trace FarRed (Thermo Fisher Scientific, Waltham, MA, USA) according to manufacturer’s instructions. Primary leukemia cells were incubated with effector cells at the ratio of 1:1 or incubated alone for 4 h. Cells were harvested and stained with PE-anti-CD5 antibody, and the CD5-positive population in FarRed-labeled cells was determined by flow cytometry. The killing efficacy was analyzed based on the FarRed^+^CD5^+^ percentage in wells with target cells alone (% killing = 100 – (FarRed^+^CD5^+^ percentage from wells plated with effector and target cells) / (FarRed^+^CD5^+^ percentage from wells plated with target cells only) ×100). The HD T cells were incubated with NK or CAR-NK cells at a ratio of 1:1 and the FarRed^+^ percentage was analyzed at the start and after 4 h of co-incubation. The killing efficacy was analyzed based on the FarRed^+^ percentage at the start (% killing = 100 – (FarRed^+^ percentage after 4 h) / (FarRed^+^ percentage at the start) ×100).

### Cytokines analysis

CAR-NK cells were cultured at the indicated density for 24–48 h. The secreted IL-15 in the cell culture supernatant and serum samples was detected using ELISA kits specific for human IL-15 (QuantiCyto, Shenzhen, China). Cell supernatants from the cytolysis assay and mouse serum were collected and stored at -80 °C. A single thaw was performed. Detection was performed using the human TNF-α ELISA Kit (QuantiCyto, Shenzhen, China) and human IFN-γ ELISA Kit (QuantiCyto, Shenzhen, China). The specific procedures were performed according to the manufacturer’s instructions.

### GCV-mediated depletion of HSV-TK expressing CAR-NK cells

CAR-NK cells expressing HSV-TK or not were seeded at an appropriate density and treated with GCV or vehicle. Cells were counted using trypan blue exclusion and the CAR positive proportion was analyzed by flow cytometry at the indicated time points.

### Jurkat-derived xenograft murine models

Six-week-old female NCG (NOD-Prkdc^em26Cd52^IL2rg^em26Cd22^/Nju) mice were purchased from GemPharmatech (Nanjing, China). Mice were maintained under standardized pathogen free conditions with adequate access to food and water and were approved by the Institutional Animal Care and Use Committee (IACUC) of Tongji Hospital (Approval ID: T1-2024-08-074). NCG mice were engrafted with 1 × 10^6^ Jurkat-luci cells via tail vein injection on day 0. At day 4 post tumor cell application, mice were randomly divided into four groups according to the average radiance of bioluminescent imaging (BLI): CAR-NK, CAR-T, NK, and PBS. Each group was injected with 2 × 10^6^ CAR-T or 1 × 10^7^ CAR-NK/ NK cells via the tail vein, and the PBS-treated groups were served as controls. One week later, mice infused with CAR-NK and NK cells were re-administered equal amounts of effector cells. Leukemic burden was evaluated using BLI, and body mass and survival were monitored regularly. The percentage of effector and leukemia cells in the peripheral blood was analyzed by flow cytometry on a weekly basis.

At the indicated time points, mice were euthanized, and their livers and spleens were isolated. The tissue specimens were fixed in 4% paraformaldehyde (PFA) solution at 4 °C overnight, followed by sequential dehydration in graded sucrose solutions. The specimens were then embedded in optimal cutting temperature (OCT) compound, snap-frozen, and cryosectioned at 5–10 μm thickness using a cryostat. These slices were used for immunofluorescence staining. During immunofluorescence staining, the sections were permeabilized with 0.1% Triton X-100 in PBS for 10 min and blocked with 5% bovine serum albumin (BSA) in PBS for 1 h at room temperature. Advanced decalcification is required for bone samples. Anti-human CD5 antibody (A9557, ABclonal, China), anti-human CD56 antibody (ab75813, Abcam, China), and anti-human CD3 antibody (A25012, ABclonal, China) were applied and incubated overnight at 4 °C. The next day, sections were washed with PBS and incubated with the secondary antibodies for 1 h at room temperature. Nuclei were counterstained with DAPI for 5 min, followed by three washes with PBS. The sections were mounted with antifade mounting medium and visualized under a fluorescence microscope. Quantitative analysis was performed using the ImageJ software to assess the proportion of positive cells or fluorescence intensity.

### Graphs and statistical analysis

Graphs and data analyses were performed using GraphPad Prism Software version 8.3.0. All data are presented as mean ± standard deviation (SD). One-way analysis of variance, two-way analysis of variance, Student’s t-test, or log-rank test were used as appropriate to compare significant differences. Survival probabilities were calculated using the Kaplan–Meier method. *P*-values are represented as either not significant (ns), *p < 0.05, ** *p* < 0.01, *** *p* < 0.001, or **** *p* < 0.0001.

## Results

### Ex vivo expansion of PB-derived CAR-NK cells with engineered K562 feeders

**Fig. 1:**
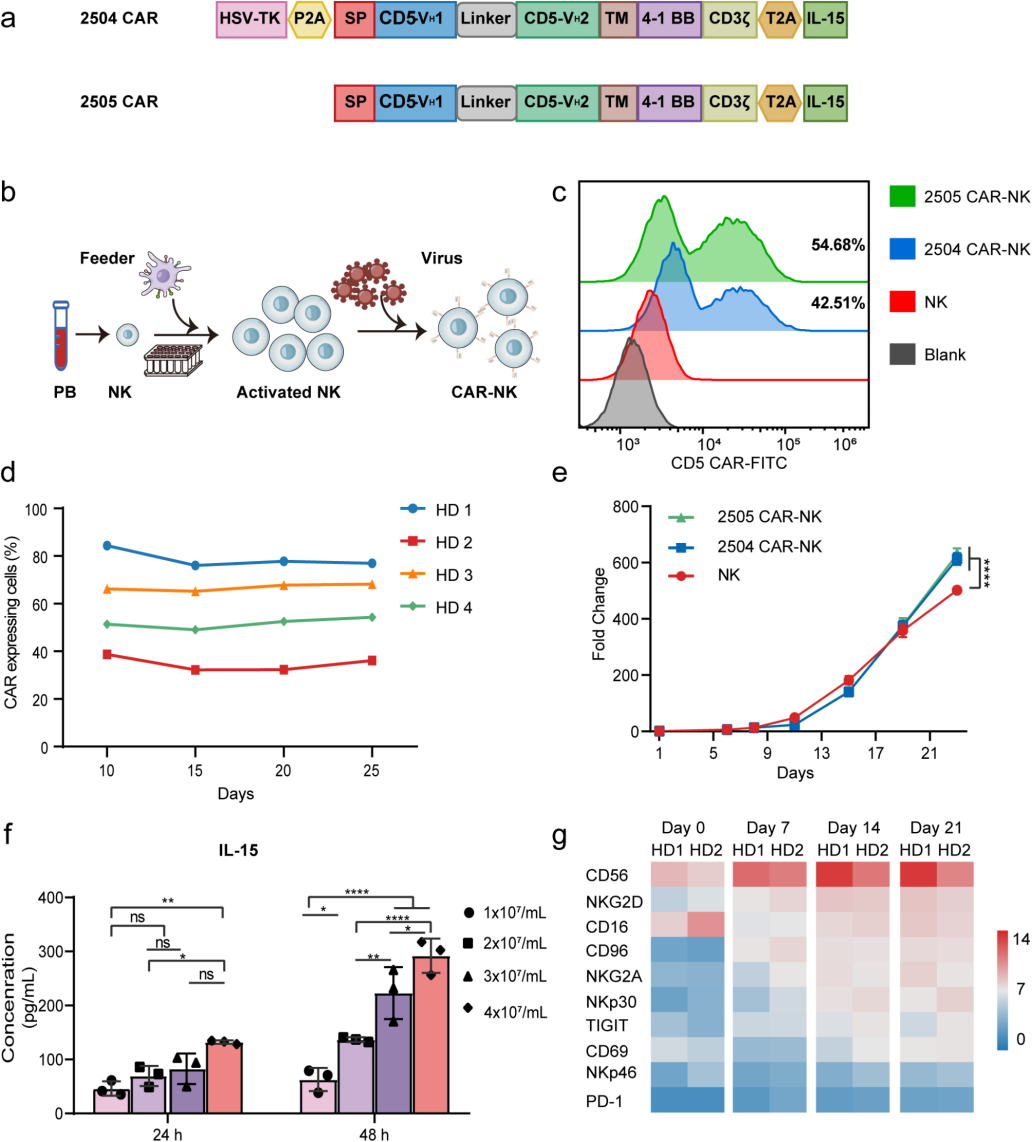
Manufacturing of PB-derived CD5 CAR-NK cells with engineered K562 feeders. (**a**) Schematic diagram illustrating the CD5 CAR constructs with or without the HSV-TK suicide gene system (2504 and 2505, respectively). (**b**) Procedure for generating CD5 CAR-NK cells from PB. (**c**) Representative flow cytometry analysis showing the transduction efficiency of CAR-NK cells. (**d**) Variability in CAR transduction efficiency among NK cells from different donors and its temporal changes. (**e**) Expansion of CAR-NK cells ex vivo. (**f**) IL-15 secretion profile of CAR-NK cells. (**g**) Longitudinal analysis of the activating and inhibitory receptor profiles of CAR-NK cells from two healthy donors. The mean fluorescence intensity (MFI) of each marker was normalized using side scatter (SSC) values, and the square root of the resulting values was plotted as a heatmap

Two CD5-targeting CAR constructs, designated as 2504 and 2505, were engineered. Both constructs incorporate two fully human VH domains, a CD8-derived hinge and transmembrane region, a 4-1BB costimulatory domain, a CD3ζ signaling domain, and IL-15. Additionally, construct 2504 carries an HSV-TK safety switch (Fig. 1a). NK cells derived from the peripheral blood of heathy donors were activated by feeder cells and transduced with CD5 CAR-containing baboon enveloped virus (BaEV) (Fig. 1b). This approach successfully generated CAR-NK cells with transduction efficiencies of 42.51% and 54.68%. (Fig. 1c). Although the efficiency of transgene integration ranged between 30 and 80% among different donors, sustained CAR expression was observed in the NK cells (Fig. 1d). CAR transduction did not adversely affect the expansion of NK cells and CAR-NK cells exhibited a relative growth advantage compared to un-transduced NK cells after three weeks (Fig. 1e). The secretion of IL-15, which is associated with CAR expression, was detected and showed progressive accumulation in correlation with both temporal progression and increased cell density (Fig. 1f). Continuous monitoring of the CAR-NK cell immunophenotype revealed that CD56 maintained the highest expression intensity and continued to increase. Receptors associated with NK cell activation, such as CD16, NKG2D, NKp30, and CD69, were also progressively upregulated. Some inhibitory receptors, such as CD96, NKG2A, and TIGIT are expressed concomitantly with cell activation. However, PD-1 expression was undetectable in CAR-NK cells (Fig. 1g).

### CD5 CAR-NK cells demonstrate superior efficacy in eliminating CD5-positive tumor cell lines in vitro

**Fig. 2:**
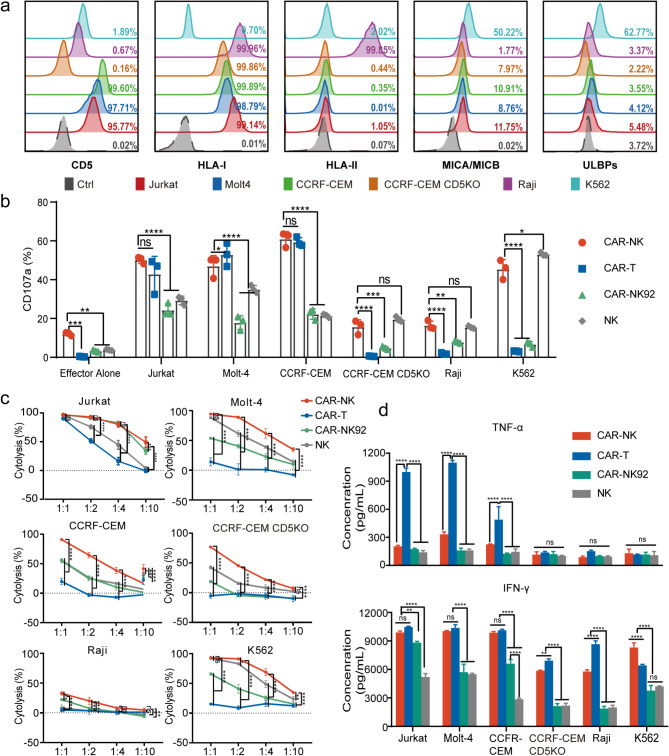
CD5 CAR-NK cells showed superior efficacy in eliminating CD5-positive tumor cell lines in vitro. (**a**) Flow cytometric analysis of surface expression levels of CD5, HLA-I, HLA-II, MICA/MICB, and ULBPs across multiple cell lines, normalized to isotype controls. (**b**) Degranulation analysis of CAR-NK, CAR-T, CAR-NK92, and NK cells. Data was presented as the mean ± SD from three independent donors (*n* = 3). (**c**) Cytolysis assay of CAR-NK, CAR-T, CAR-NK92 and NK cells. Data represent the mean ± SD for three donors. (**d**) Quantitative analysis of TNF-α and IFN-γ secretion by CAR-NK, CAR-T, CAR-NK92, and NK cells following co-culture with the specified target cells

Six cell lines were used to evaluate the cytotoxic effects of CD5 CAR-NK, CAR-NK92, CAR-T and NK cells in vitro. Three of these cell lines, derived from T-cell leukemia/lymphoma (Jurkat, Molt4, and CCRF-CEM), exhibited robust CD5 expression. Raji cells and CD5 knockout CCRF-CEM (CD5KO-CCRF) were used as negative controls, as no CD5 expression was detected in these cells. The K562 cell line, which also lacks CD5 expression, was used as a positive control because of its susceptibility to NK cells (Fig. 2a). Unlike T cells, NK cell activity is regulated by the balance of signals from inhibitory and activating receptors on their surface. Therefore, ligands for inhibitory signals, such as human leukocyte antigen-I (HLA-I) and HLA-II, and ligands for activating signals, such as MHC class I chain-related genes A and B (MICA/MICB) and UL16-binding proteins (ULBPs), were also evaluated in these cell lines. As expected, K562 cells exhibited the highest expression of activating ligands and the lowest expression of inhibitory ligands, whereas Raji cells showed the strongest expression of both HLA-I and HLA-II (Fig. 2a).

Cytotoxic granule production by effector cells was evaluated using a degranulation assay, with CD107a expression serving as a marker of degranulation activity. Upon stimulation with CD5-positive target cells, CD5 CAR-NK and CAR-T cells exhibited comparable levels of CD107a degranulation, which were significantly higher than those observed in CAR-NK92 and NK cells. However, when co-cultured with CD5-negative cells, only CAR-NK and NK cells displayed a significant increase in CD107a release (Fig. 2b). This reflects the inherent anti-tumor cytotoxicity of NK cells. The cytolytic capability of CAR-NK cells was demonstrated by their ability to eliminate CD5-positive malignant cell lines at a low effector-to-target ratio (1:10) within a short 4-hour period, outperforming NK, CAR-NK92, and CAR-T cells (Fig. 2c). In CD5-negative cell lines, CAR-NK and NK cells exhibited the strongest lytic activity owing to their CAR-independent cytotoxic effects, particularly in K562 cells. In contrast, neither NK nor CAR-NK cells showed significant lysis of Raji cells, which do not express CD5 but have high levels of HLA-I and HLA-II expression (Fig. 2c). Furthermore, the production of TNF-α and IFN-γ confirmed the specificity of CAR-NK and CAR-T cells, with CAR-NK cells exhibiting a preference for secreting IFN-γ over TNF-α upon activation (Fig. 2d).

### CD5 CAR-NK cells exhibit enhanced cytotoxic activity against CD5-positive primary leukemia cells in vitro

**Fig. 3:**
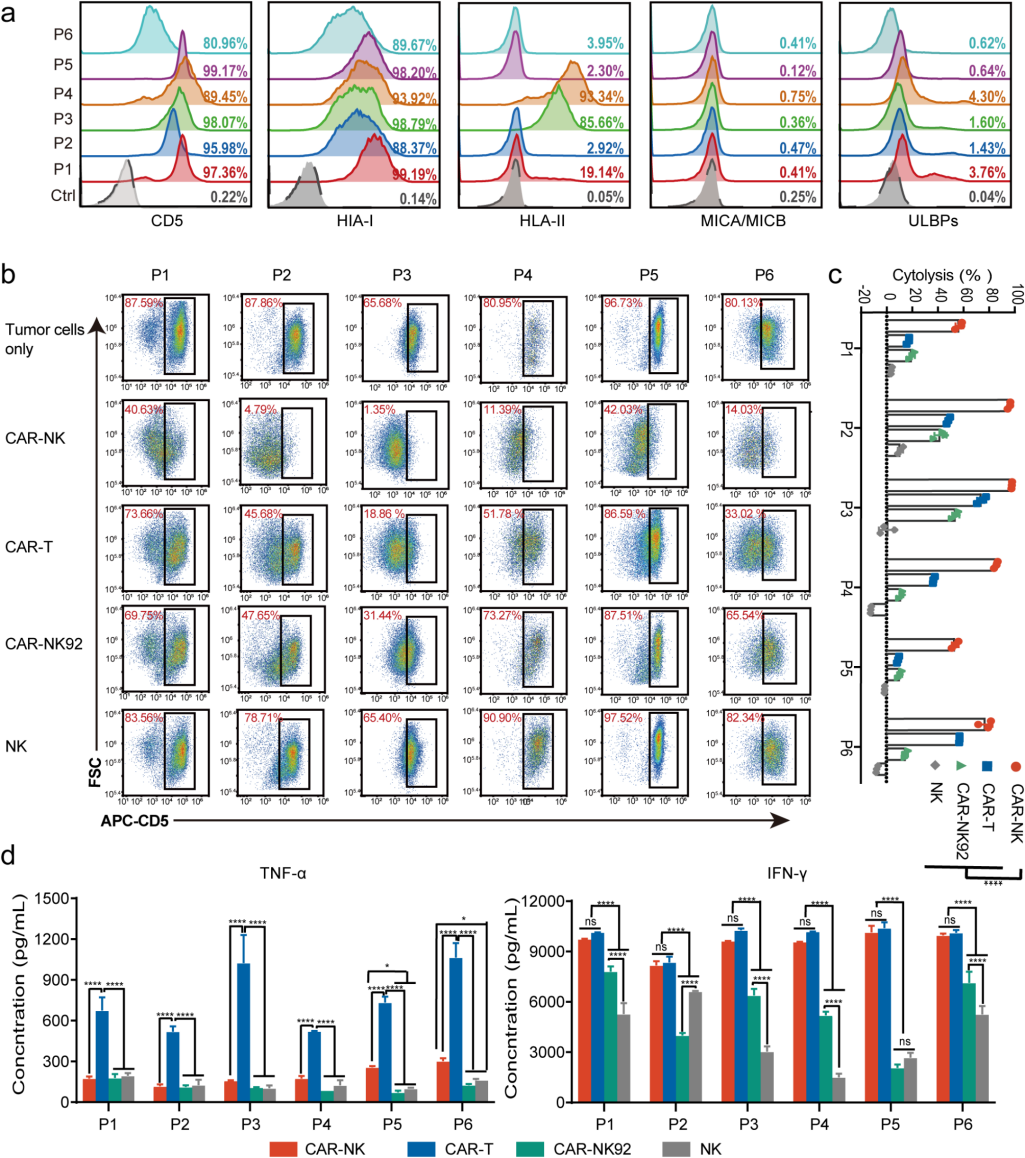
CD5 CAR-NK cells exhibit enhanced cytotoxic activity against CD5-positive primary leukemia cells in vitro. (**a**) The surface expression levels of CD5, HLA-I, HLA-II, MICA/MICB, and ULBPs in six patient samples were measured by flow cytometry. (**b**) Representative flow cytometry plots showing the percentage of CD5-positive cells in each patient sample, either cultured alone or co-cultured with effector cells for 4 h. (**c**) Cytotoxicity of effector cells, as determined by the lysis of primary tumor cells. (**d**) Quantification of TNF-α and IFN-γ secretion by CAR-NK, CAR-T, CAR-NK92 and NK cells after co-culture with primary tumor cells

To further verify the cytotoxicity of CAR-NK cells against primary leukemic blasts, PBMCs were collected from six patients for further analysis. The characteristics of patients are provided in Supplemental Table S1. Tumor cells from each patient were assessed for target antigen expression, which confirmed the high expression of CD5. Additionally, the expression of activating and inhibitory ligands was analyzed by flow cytometry (Fig. 3a). Due to disease variability, only the tumor cells from two CLL patients (P3 and P4) were positive for HLA-II. All primary tumor cells exhibited either absent or low expression of activating ligands. After brief co-culture with CAR-NK, CAR-T, CAR-NK92, and NK cells, CAR-NK cells significantly cleared all primary tumor cells, followed by CAR-T and CAR-NK92 cells. NK cells showed a notable deficiency in recognizing and killing primary tumor cells from patients (Fig. 3b-c). Cytokines, particularly IFN-γ, were released in large quantities concomitant with the functional activity of CAR-NK cells (Fig. 3d). The results indicated that CAR-NK cells were capable of recognizing CD5-positive primary tumor cells, that had developed resistance to NK cells. Moreover, CD5 CAR-NK cells exhibited significantly greater cytotoxic efficacy than CAR-NK92 and CAR-T cells.

### CD5 CAR-NK cells demonstrate more pronounced antileukemic effects in cell line-derived xenograft (CDX) mouse model

**Fig. 4:**
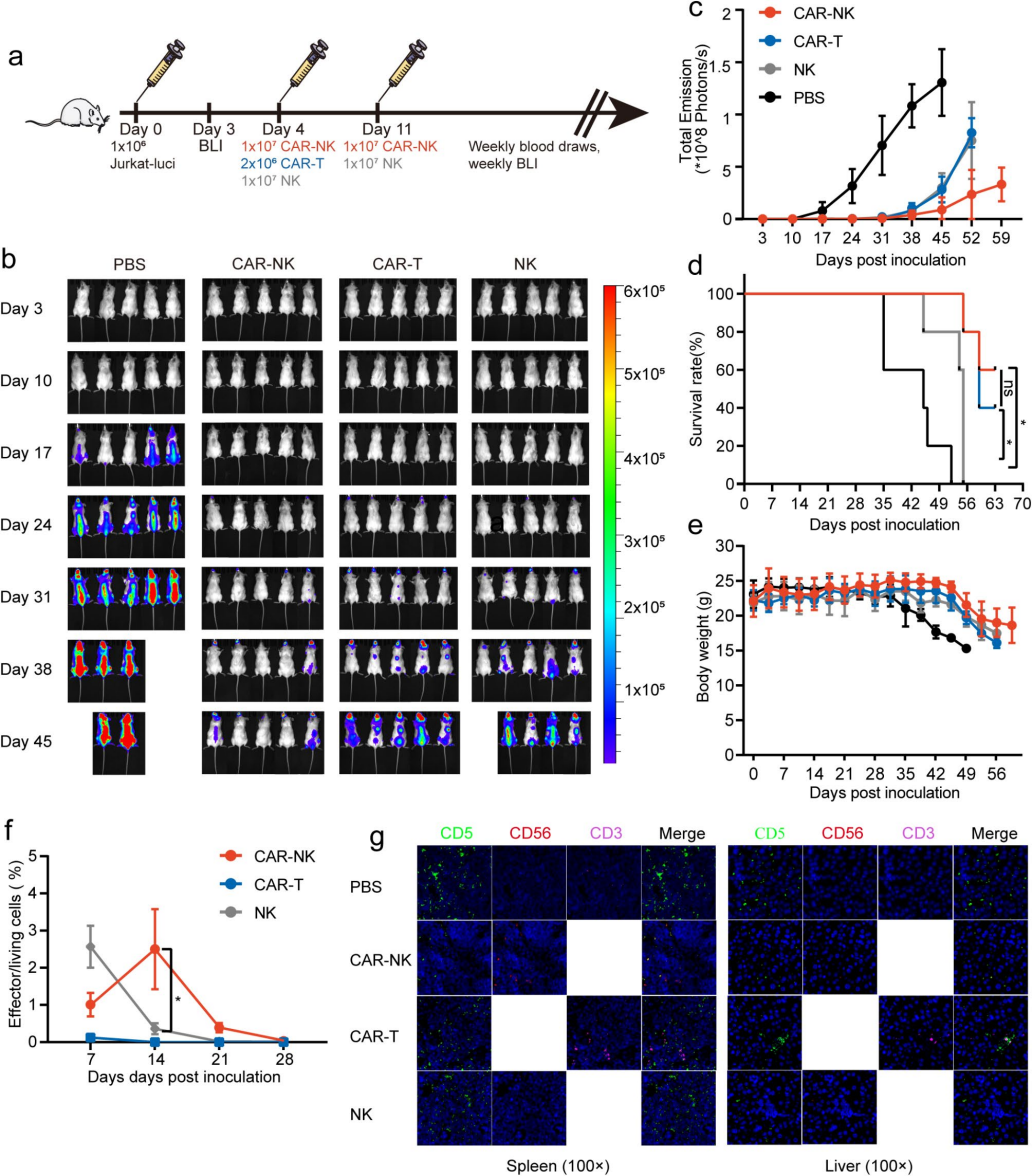
The superior anti-tumor activity of CD5 CAR-NK cells in vivo. (**a**) Schematic representation of the in vivo experimental timeline. (**b**) Tumor growth and staging monitored by BLI. (**c**) Average radiance quantification for each group at the indicated time points. The results are presented as mean ± SD (*n* = 5). (**d**) Kaplan–Meier survival curve for each group. Data are presented as mean ± SD (*n* = 5). (**e**) Body weight curve for each group. The results are presented as mean ± SD (*n* = 5). (**f**) Percentage of CAR-NK, CAR-T, and NK cells in the peripheral blood of the mice (*n* = 5). (**g**) Representative images of immunofluorescence labeling of infiltrating tumor cells (CD5, green), NK/CAR-NK cells (CD56, red), or CAR-T cells (CD3, pink) in the spleen (left) and liver (right) of mice treated with CAR-NK, NK, CAR-T cells, or control

Using a Jurkat-derived xenograft mouse model, we investigated the antileukemic effects of CD5 CAR-NK, CAR-T and NK cells in vivo. Effector cells were administered starting on day 4 post tumor cells injection, with dosage and frequency for each cell type personalized based on clinical experience (Fig. 4a). Despite the single dose of CAR-NK cells being 10 times that of CAR-T cells, CAR-NK cells demonstrated a favorable safety profile. One week after the initial administration, NK and CAR-NK cells were re-administered to reinforce their therapeutic effects. Longitudinal bioluminescence imaging (BLI) monitoring demonstrated that CAR-NK, CAR-T, and NK cells all exhibited anti-tumor efficacy in vivo, with CAR-NK-treated mice showing significantly reduced tumor burden compared to CAR-T and NK cell treatment groups at matched time points (Fig. 4b-c). Kaplan-Meier survival analysis demonstrated that the administration of CAR-NK, CAR-T, and NK cells significantly extended the overall survival of tumor-bearing mice compared to untreated controls. The survival rates between CAR-T and CAR-NK cell treatment groups showed no statistically significant difference at this dosage level. However, mice receiving CAR-NK cell therapy demonstrated superior long-term survival outcomes than receiving NK cells (Fig. 4d). IL-15 remained below detectable thresholds in all serum samples across all timepoints. CAR-NK cell-treated subjects exhibited significantly elevated serum concentrations of IFN-γ and TNF-α at day 14 post-administration (Supplemental Fig. 1). Statistical analysis revealed no significant differences in body weight between the experimental groups (Fig. 4e). Flow cytometric analysis was performed to quantify effector cells in peripheral blood samples from different groups, specifically detecting CD56^+^ cells for NK and CAR-NK cells, and CD3^+^ cells for CAR-T cells (Fig. 4f). While all three cell populations (CAR-NK, CAR-T, and NK cells) exhibited low relative percentages and approached undetectable levels by day 28, CAR-NK cells demonstrated transient expansion during the initial two-week period, distinguishing them from the steady decline observed in the other two cell types (Fig. 4f). On day 45, the animals were humanely euthanized and tissues were harvested for analysis of tumor burden and immune cell infiltration. Enhanced CAR-NK cell infiltration and reduced tumor cell presence were observed in the liver and spleen of mice receiving CAR-NK cell therapy. Analysis of hepatic and splenic tissues revealed robust CAR-NK cell infiltration accompanied by marked reduction in tumor burden (Fig. 4g). Collectively, these findings suggest that CD5-targeted CAR-NK cell therapy holds considerable promise as a therapeutic approach for CD5-expressing hematologic malignancies.

### The on-target off-tumor toxicity of CD5 CAR-NK cells can be mitigated through GCV-mediated switch-off

**Fig. 5:**
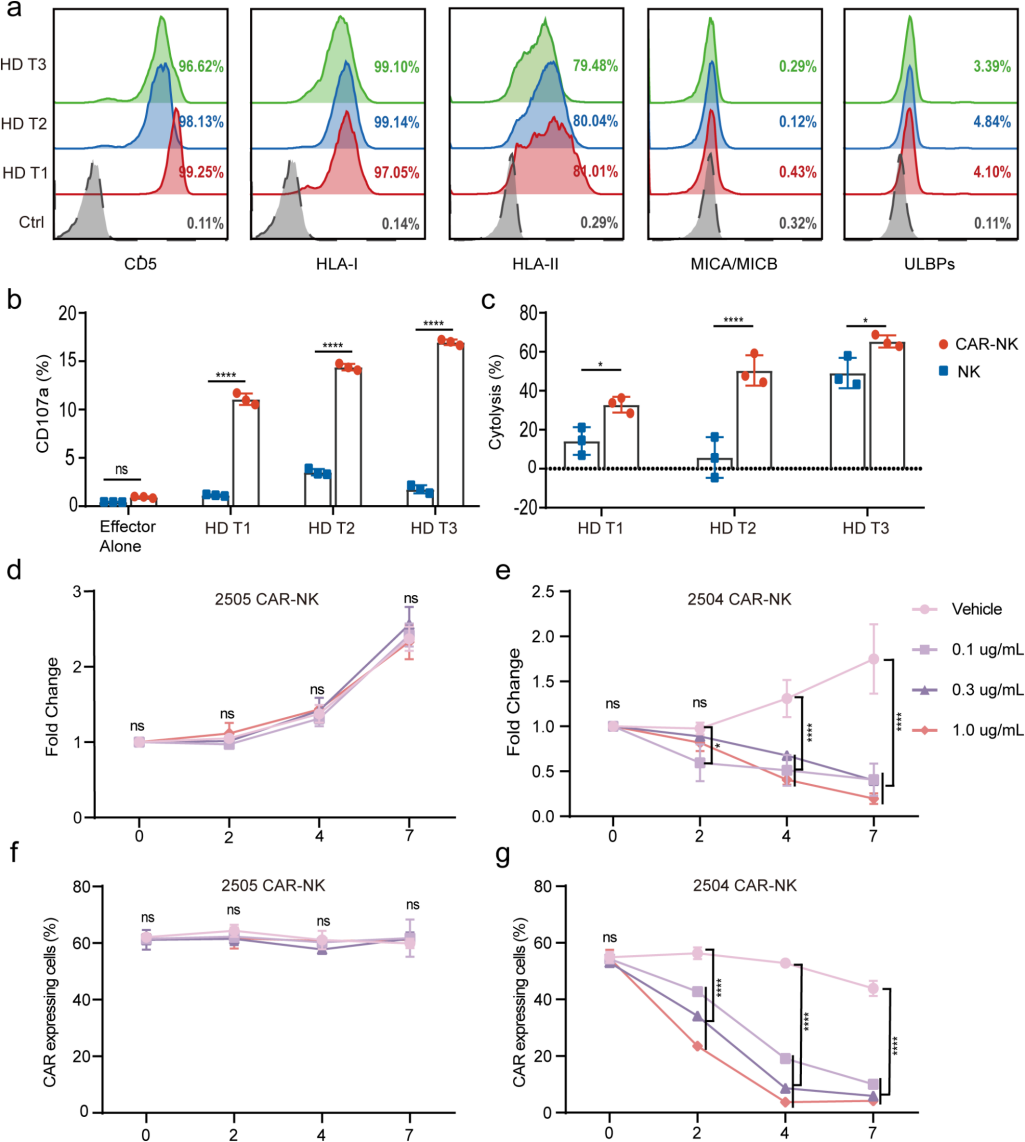
The HSV-TK system can promptly shut down the expression of CAR-NK cells in vitro. (**a**) Surface expression of CD5, HLA-I, HLA-II, MICA/MICB, and ULBPs in T cells from three healthy donors as measured by flow cytometry. (**b**) Degranulation of NK and CD5 CAR-NK cells, as measured by CD107a surface expression following stimulation with healthy donor (HD)-derived T cells. NK cells and CD5 CAR-NK cells isolated from HDs were co-cultured with allogeneic HD-derived CD5-positive T cells at a 1:1 effector-to-target (E: T) ratio. Degranulation activity was quantified using flow cytometry after 4 h of co-culture. Data represent the mean ± SD of CD107a^+^ frequencies within the CD56^+^ NK/CAR-NK cell populations across three independent donors, ensuring specificity to NK cell responses by excluding non-NK lineages through pre-gating strategies. (**c**) Cytolysis of NK and CD5 CAR-NK cells by HD-derived T cells. The data are presented as the mean ± SD for three donors. (**d**) Proliferation of CAR-NK cells without HSV-TK (2505) in the presence of GCV or the vehicle control. (**e**) Proliferation of CAR-NK cells with HSV-TK (2504) in the presence of GCV or vehicle control. (**f**) Changes in the percentage of CAR-expressing cells without HSV-TK (2505) after treatment with GCV or vehicle control. (**g**) Changes in the percentage of CAR-expressing cells with HSV-TK (2504) after treatment with GCV or vehicle control

The therapeutic application of CD5 CAR-NK cells necessitates thorough safety evaluation, as the CD5 antigen is endogenously expressed in normal T lymphocytes, raising potential concerns about on-target, off-tumor effects. To evaluates the potential side effects of CD5 CAR-NK cells on normal T cells, T cells from three healthy donors were isolated and assessed for the expression of CD5 along with activating and inhibitory ligands. As expected, nearly all activated T cells expressed CD5, HLA-I and HLA-II but lacked the expression of MICA/MICB and ULBPs (Fig. 5a). In the allogeneic co-culture system, T cells induced moderate activation of CAR-NK cells, as evidenced by enhanced CD107a expression, indicating significant degranulation of CAR-NK cells (Fig. 5b). Additionally, CD5 CAR-NK cells exhibited varying levels of cytotoxicity against allogeneic T cells (Fig. 5c).

To enable the temporal control of CAR expression on NK cells, we employed the FDA-approved drug ganciclovir (GCV) as a switch-off. The suicide gene HSV-TK converts the non-toxic prodrug GCV into a cytotoxic metabolite that disrupts cellular function, enabling targeted elimination of cells expressing this system. Various concentrations of GCV were administered to CAR-NK cells expressing either the HSV-TK transgene. The results demonstrated that the proliferation of 2505 CAR-NK cells remained unaffected by GCV treatment, whereas the number of 2504 CAR-NK cells decreased in a dose- and time-dependent manner (Fig. 5d-e). Simultaneously, the percentage of CAR-expressing cells was monitored: CAR expression in 2505 CAR-NK cells remained stable, whereas CAR-positive cells in the 2504 CAR-NK group showed a continuous decline (Fig. 5f-g). These data demonstrate that CD5 CAR-NK cells exhibit unintended cytotoxicity against normal T lymphocytes. However, this off-target activity can be effectively mitigated through GCV-mediated selective depletion of CAR-NK cells.

## Discussion

CD5 is a promising therapeutic target for T-cell leukemia and lymphomas. However, their constitutive expression on normal T cells raises concerns that CAR-T cells targeting CD5 may induce fratricide and T-cell aplasia [19]. Antigen ablation of T cells enables the generation of CAR-T cells, but additional genetic modifications of T cells can lead to unpredictable outcomes. Previous studies have shown that CD7-negative T cells exhibit marked activation of autoimmune-related pathways following anti-CD7 CAR-T cell therapy [23]. An investigator-initiated trial (NCT04767308) at our center demonstrated that patients receiving CD5-knockout CD5 CAR-T cell therapy experienced severe adverse events including severe rash [24] and recurrent infections, which were suspected to be associated with prolonged failure to recover CD5-positive T cells. Given that NK cells naturally lack CD5, CD5 CAR-NK cells are a viable option for immunotherapy against T-cell malignancies.

Recent studies have demonstrated that primary CAR-NK cells are both safe and highly effective [18, 25]. However, research focusing specifically on CD5-targeted CAR-NK cells remain limited. In this study we found that primary CD5 CAR-NK cells exhibited stable CAR expression and maintained a high proliferation rate ex vivo. Furthermore, CD5 CAR-NK cells efficiently eliminated CD5-positive cell lines and primary tumor cells in vitro, prolonged the survival of tumor-bearing mice, and reduced tumor infiltration without causing observable side effects. Constitutive secretion of IL-15 can cause severe toxicity in animal models [26]. In this study, CAR expression in tandem with IL-15 secretion did not cause detectable toxicity, which may be attributed to the low concentration of IL-15.

In this study, CAR-NK cells were generated from peripheral blood-derived NK cells. Compared to CB, induced pluripotent stem cells (iPSC) [27] and hematopoietic stem and progenitor cells (HPSC)-derived NK cells, peripheral blood-derived NK cells exhibit a more mature phenotype and enhanced effector functions [28]. Irradiated CAR-NK92 cells which lack proliferative capacity are unlikely to have sustained effects [29]. We conducted a comparative study of CD5 CAR-NK, CAR-NK92, CAR-T cells, and NK cells. CD5 CAR-NK92 cells exhibited mild antileukemic activity in vitro, even when not irradiated. And CD5 CAR-NK cells demonstrated rapid and superior anti-tumor efficacy compared to NK, CAR-T and CAR-NK92 cells, both in vitro and in vivo. Furthermore, CAR-NK cells retained robust cytolytic activity against primary tumor cells that were originally resistant to NK cell-mediated killing.

Previous studies have performed head-to-head comparisons between CAR-NK cells and CAR-T cells targeting CD19 and CD123, and reported contrasting conclusions [30, 31]. Egli et al. found that CD19 CAR-T cells exhibited higher CAR-mediated effector functions and achieved stronger anti-tumor effects than CD19 CAR-NK cells [30]. Caruso et al. demonstrated that CD123 CAR-NK cells showed significant anti-leukemia activity with limited on-target off-tumor toxicity compared with CAR-T cells [31]. The differences in conclusions from these studies can be attributed to variations in dosing regimens and administration frequencies, as well as the distinct targets investigated in each study. Given the limited lifespan of NK cells, repeated administration has been shown to preserve the metabolic functionality of CAR-NK cells [32]. Our infusion protocol leverages the high safety and tolerance of CAR-NK cells and is representative of the doses used in human CAR-T and CAR-NK cell applications [18, 24].

It is critical that “live drugs”, such as CAR-T or CAR-NK cells, be engineered with safety switches to allow intervention in case of adverse events. Although previous studies have demonstrated a good safety profile for CD19 CAR-NK cells, the adverse effects of CD5 CAR-T cells, particularly T cell aplasia, have raised concerns [10, 33]. We previously utilized a truncated EGFR switch for CD5 CAR-T cells, but this system relies on Cetuximab and antibody-dependent cell cytotoxicity (ADCC) mediated by NK cells [19, 24]. Given the concerns regarding reduced efficacy with decreasing NK cell numbers, we implemented a simpler system controlled by small molecules for CAR-NK cells. The HSV-TK gene is another off-switch system [34]. It can be stably expressed without affecting the NK cell viability and proliferation. Administration of GCV at low concentrations resulted in selective elimination of CAR-NK cells expressing the suicide gene construct with high efficiency. However, the immunogenicity of the HSV-TK/GCV system poses a potential limitation in clinical applications, as viral-derived proteins such as HSV-TK may elicit host immune responses against engineered cells. Genetic modifications to reduces immunogenic splice variants [35], humanized suicide gene replacement, transient mRNA-based CAR-NK systems and specific immunosuppression [36] could be used to improve this system.

In conclusion, our study provides proof-of-principle for the treatment of CD5-positive malignancies by leveraging the natural advantages of NK cells. Peripheral blood-derived CD5 CAR-NK cells exhibit potent anticancer activity both in vitro and in vivo at safe doses. This approach offers a promising avenue for ‘off-the-shelf’ cell therapy for T-cell malignancies.

## Electronic supplementary material

Below is the link to the electronic supplementary material.

Supplementary Material 1

## Data Availability

Data supporting the findings of this study are available from the corresponding author upon reasonable request.
