# Supplementary material for: Engineering CD5-targeting CAR-NK cells from peripheral blood for the treatment of CD5-positive hematological malignancies
Source: J Transl Med. 2025 Apr 8;23:409. doi: 10.1186/s12967-025-06432-3 (PMC11980226; doi:10.1186/s12967-025-06432-3)
Supplement: Supplementary file 1 — Supplementary Material 1 [file 12967_2025_6432_MOESM1_ESM.docx]

**Table S1: Detail information of each patient**

| **Patient ID** | **Gender** | **Age** | **Diagnosis** | **Proportion of blast cells** | **Immunophenotyping of tumor cells** | **Mutated gene profile** | **Molecular genetics / cytogenetics** |
| --- | --- | --- | --- | --- | --- | --- | --- |
| P1 | F | 15 | T-ALL | 61.6% | CD7^dim^ CD5^+^ HLA-DR^-^ CD19^-^ CD33^-^ | *FBXW7*, *USP7*, *KLF2*， *KMT2D*, *RB1* | *STIL-TAL1* fusion gene |
| P2 | M | 15 | T-ALL | 56.7% | CD45^+^ TdT^+^ CD7^bri^ CD5^bri^ CD3^dim^ CD8^dim^ | *PHF6*, *ARID1A* | *TCRBA*, *TCRBC*, *TCRGA* rearrangement (+) |
| P3 | M | 66 | CLL | 98.0% | CD19^+^ CD20^+^ CD5^+^ CD23^+^ CD200^+^ CD43^+^ CD79b^dim+^ Bcl-2^+^ | *BCL2*, *IGLL5*, *PRF1*, *PRKCD*, *NLRP1*, *RAD51*, *APC* | NA |
| P4 | F | 53 | CLL | 68.6% | CD5^+^ CD10^-^ Ki67^-^ | NA | Trisomy 12; *IGH* rearrangement (+) |
| P5 | F | 38 | T-LBL | 87.9% | CD45^+^ CD7^bri^ cCD3^+^ CD5^+^ CD99^bri^ CD8^-^ CD3^-^ | NA | NA |
| P6 | F | 62 | T-LBL | 83.1% | CD45^+^ CD7^bri^ CD5^+^ cCD3^+^ CD99^bri^ CD19^dim^ CD34^-^ CD4^-^ CD8^-^ CD56^-^ CD16^-^ CD20^-^ CD138^-^ HLA-DR^-^ | NA | NA |

F, female; M, male; T-ALL, T-cell acute lymphoblastic leukemia; CLL, chronic lymphocytic leukemia; T-LBL, T cell lymphoblastic lymphoma.

**Figure S1 *In vivo* cytokine secretion profile of NK, CAR-T and CAR-NK cells**


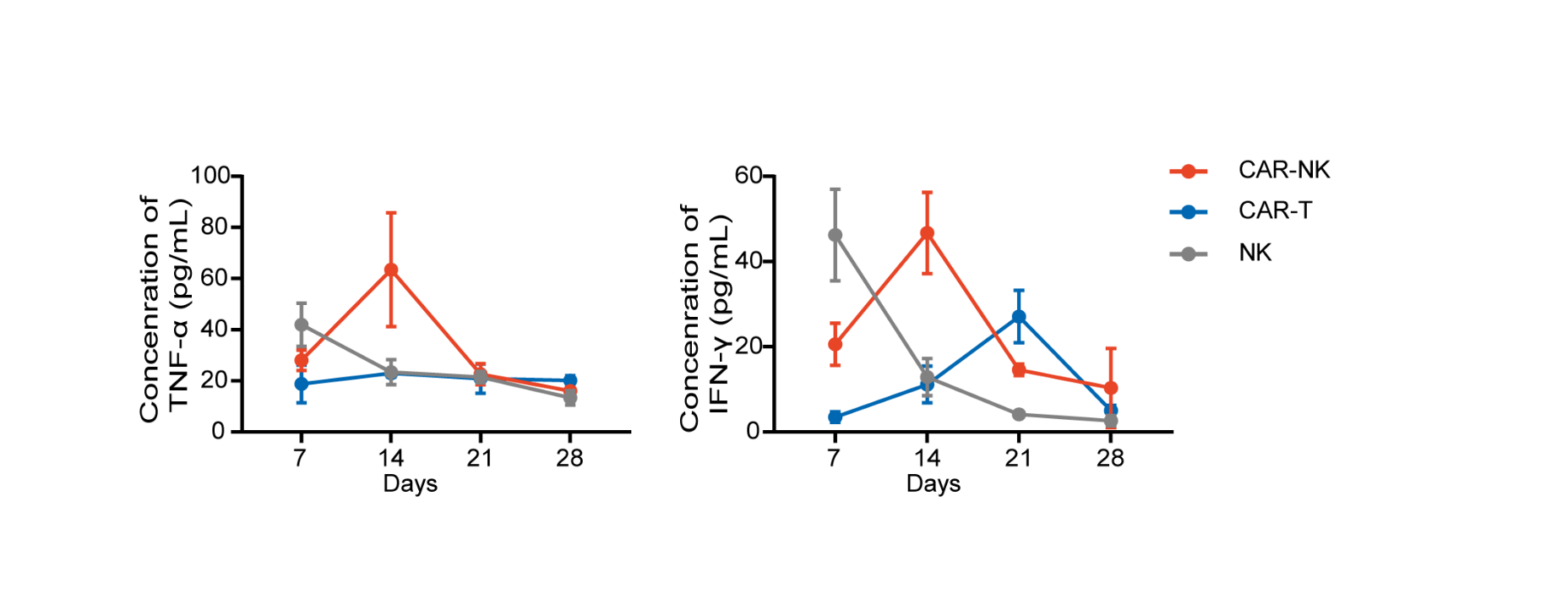


The serum concentrations of TNF-α and IFN-γ at specified time points in treated mice were quantitatively assessed using enzyme-linked immunosorbent assay (ELISA).
